# Thinking About Denial

**DOI:** 10.1093/hwj/dbx040

**Published:** 2017-08-23

**Authors:** Catherine Hall, Daniel Pick

**Affiliations:** University College London Birkbeck University of London

## Abstract

This essay considers the frequent and varied uses of ‘denial’ in modern political discourse, suggests the specific psychoanalytic meanings the term has acquired and asks how useful this Freudian concept may be for historians. It notes the debates among historians over the uses of psychoanalysis, but argues that concepts such as ‘denial’, ‘disavowal’, ‘splitting’ and ‘negation’ can help us to understand both individual and group behaviour. The authors dwell, especially, on ‘disavowal’ and argue it can provide a particularly useful basis for exploring how and why states of knowing and not knowing co-exist. Historical examples are utilized to explore these states of mind: most briefly, a fragment from a report about the war criminals, produced by an American psychiatrist at the Nuremberg Trial; at greater length, the political arguments and historical writings of an eighteenth-century slave-owner; and finally, a case in a borough of London in the late-twentieth-century, where the neglect, abuse and murder of a child was shockingly ‘missed’ by a succession of social agencies and individuals, who had evidence of the violence available to them.

## NUREMBERG 1946

One evening in May 1946, during the trial of the major war criminals at the International Military Tribunal, the American psychiatrist Leon Goldensohn encountered Hermann Göring in his cell. The prisoner was smoking his long Bavarian hunting pipe. This was the former *Reichsmarschall*, a man who was for many years an extremely powerful operator in the Nazi Party, centrally involved in German military planning, the commanding figure at the Luftwaffe, and the acolyte whom Hitler had appointed as his successor and deputy. According to Goldensohn, he appeared subdued and low, although on seeing the visitor at the door, the accused ‘smiled forcibly in an attempt to appear cheerful’. Goldensohn asked if he might be feeling depressed about something. Whereupon Göring looked at the wall and replied:

Well, this sciatica has got me down a little bit, but I must admit that in general I don't feel as cheerful as I might. I don't understand it myself…. You know, *I spend a good deal of my time in fantasy*. For example, when things get dull or unpleasant in the courtroom, *I can close my eyes* behind my dark glasses and I practically live in the past. I think of the many pleasant times that I had. For example, I think of the frequent large parties I had in Karin Hall or of my popularity among the German people, which gives me great pleasure and satisfaction. I am sure that I will go down in history as the man who did much for the German people. This trial is a political trial, not a criminal one. If there were criminal things perpetrated by the party, or the SS, or even the army, as it is charged, *I certainly had nothing to do with them*. It is true that my position as second-in-command politically next to Hitler makes such a statement seem ridiculous. Maybe *I closed my eyes to the real meaning of what was going on in Germany*, but it was always for the benefit of the common people that I strived.^1^ [Emphasis added]

Göring had no illusions that he would be acquitted. He did not believe denial, in the legal sense, would save him. He even suggests some insight into his own predilection for fantasy, a world of ‘dark glasses’, behind which to ‘close my eyes’. Yet his response is striking, even stupefying, perhaps, for its banal evasion of the deeper import of the question. His refusal to recognize the central reality of vast Nazi war crimes and the part he played in them provides a dramatic example of denial, the concept so much in the news of late, that we hope to investigate in this essay and in the accompanying featured articles in this issue.^2^

We focus on the Freudian idea of denial in relation to the politics of remembering, forgetting and disavowal. How useful is the concept of denial for historians? It was Freud’s dynamic model of the mind and insistence on the significance of unconscious processes, which brought questions of denial to new prominence. We aim briefly to identify and discuss an individual and collective example of denial, and set out the psychoanalytic meanings and implications of associated key terms. The problem of denial has a particular urgency today.

Ours is a time when statistical evidence about climate change, poverty, inequality, health and immigration is contested daily. ‘Alternative facts’ are flamboyantly conjured, at will, in defiance of all available data, not least by the current US president, and his shameless officials at the White House. Scepticism about scientific authority, a relativist view of ‘truth’, and critique of official news and mainstream cultural representations have been hijacked by climate-change deniers for grotesque and devastating political and economic purposes.^3^ It is especially relevant now to draw attention to possible psychoanalytic resources for historical and psychosocial analysis of denial, and to consider its possible, conscious and unconscious aspects.

Even the evidence of our own eyes about the relative size of two crowds can apparently be massaged away by spin-doctors.^4^ Denial and disavowal are concepts that we surely need to consider closely, with regard to the past and the present.

## JAMAICA: A SLAVE OWNER’S TESTIMONY

Edward Long, a prominent slave-owner in eighteenth-century Jamaica, left the island in 1769 on account of his ill health and returned with his family to England.^5^ He had been a leading member of the white elite, managing the plantations in Clarendon which his family had owned since Cromwell’s forces had first settled the island in the 1650s. The white colonists had been determined to establish Jamaica as a slave society from the beginning and Long’s great-grandfather, Samuel Long, was a key figure in the struggles both to secure the rights of ‘free-born Englishmen’ from the crown and to establish the enslavement of Africans as the necessary condition of that freedom.

A century later, Edward Long, who acted as secretary to his brother-in-law, the lieutenant governor, made a name for himself as one of the most opulent and intransigent members of the House of Assembly. He too was committed to upholding slavery, defending to the hilt the rights of ‘freeborn Englishmen’ against any encroachments of the executive whilst embedding the power of the masters over the enslaved. Over 600 men women and children worked in gangs on his plantations of Lucky Valley and Longville. Disciplined by white overseers and black ‘drivers’, this slave work force produced the sugar and rum that secured a very comfortable life for Long and his family.

Terror and coercion, the presence of the whip and the threat of death, were at the heart of plantation society. As the planters knew full well, ‘in countries where slavery is established, the crucial principle on which government is supported is *fear*; or a sense of that absolute coercive necessity, which, leaving no choice of action, supersedes all question of *right*’.^6^ Long kept a close eye on his human ‘stock’, mindful of their price and value, knowing that they were ‘the sinews of the plantation’, and aware too of the spectacular and terrible tortures which could be inflicted upon them at any time.

At the same time Long was engaged in defending the privileges of the white colonists. In railing against the ‘political tyranny’ that confronted them, Long adopted and adapted the language of the parliamentarians who had challenged the power of the crown in the English Revolution. In their struggle with metropolitan governments from the 1660s for control over the making of their own laws, the colonists in Jamaica had characterized themselves as ‘freeborn Englishmen’, faced with a tyrannical executive and in danger of being reduced to slavery.

Long, like many of his contemporaries, was fascinated by contemporary debates over the fixity and malleability of nature. He followed arguments about the effects of climate on human development, and discussions of the relation between the orangutan, the ‘wild man’, and the ‘savage African’. On his return to England, where he had grown up, he was deeply disturbed that the institution of slavery, long accepted as a necessary part of a prosperous nation’s business, was increasingly called into question. In 1772 the case of James Somerset received enormous coverage in the press, pamphlets and coffee houses of London. This concerned the attempted kidnapping and forcible return to the Caribbean of a man who had been brought to England as a slave but had been able to escape and live freely there.

In his celebrated judgement, Lord Mansfield, the Chief Justice of England, ruled that Somerset must be freed. Long was appalled that the rights of Englishmen to own human property could be challenged in this way, and wrote a violently polemical pamphlet arguing that slavery was a necessary institution in the colonies. Two years later he published a three-volume *History of Jamaica,* a work that he had been preparing for some time and which was given new urgency in the face of vocal criticisms of slavery. He engaged with the Enlightenment thinkers who debated the nature of man and argued, on the basis of his eyewitness experience, that black men and women were essentially different from and inferior to white. Colonial slavery, he insisted, was a necessary part of the long process of transforming African ‘savages’ into civilized people.^7^ It had taken Englishmen, he argued, centuries to become ‘civilized’. How civilized might Africans eventually become? His characteristically Enlightenment assumptions about the necessary stages of development from barbarism to civilization had the potential to undermine his insistence on the fixity of racial difference.

In the dramatic account that Long wrote of the great rebellion that took place in Jamaica in 1760, he named ‘Coromantins’ as the most dangerous of the Africans. It was they who had been responsible for the scale of the uprising. They were ‘distinguished from their brethren by their aversion to husbandry, and the martial ferocity of their disposition’. ‘Their grand enterprise … was no other than the extirpation of the entire white inhabitants.’^8^ Such an endeavour required the enactment of terrible punishments, a theatre of cruelty. He defended the need for awesome demonstrations of white supremacy and black subjection. In his description of the horrible fate of two of the ringleaders, however, a slight hint of hesitation appears: a reference to the *cruel* nature of the punishment that is ‘thought’ to be justified, rather than self-evidently justified:

Two of the St Mary’s ringleaders, Fortune and Kingston, were hung up alive in irons on a gibbet, erected in the parade of the town of Kingston. Fortune lived seven days, but Kingston survived till the ninth. The morning before the latter expired, he appeared to be convulsed from head to foot; and upon being opened, after his decease, his lungs were found adhering to the back so tightly, that it required some force to disengage them. The murders and outrages they had committed, were thought to justify this cruel punishment inflicted upon them *in terrorem* to others…

However, any weakening of will on the author’s part, or further exploration of this note of possible anxiety about the justice of white brutality, was speedily despatched by insistent reiterations of his view that Africans were unfeeling brutes: ‘they appeared to be very little affected by it themselves’, Long wrote, ‘behaving all the time with a degree of hardened insolence, and brutal insensibility’.^9^ For him, any charge that the slave system was itself an unwarranted brutality could be explained away by the brutality of Africans. He seemed to be intent on eradicating any shadow of doubt in himself, or his reader.

Long’s text is striking for its dual moral languages. Parallel psychologies and political analyses are apparent. On the one hand, a case is made, insistently, for the necessity of colonial slavery and the unfeeling and brutal nature of the slaves; on the other, eloquent descriptions are provided of the oppressions that afflicted the white planter class, the imposition of incompetent governors, ‘so horrid a group’, the outrageous ‘insolence of office’, ‘the exorbitancy of power’, the ‘violations … of liberty’ that threatened the rights of ‘freeborn Englishmen’, not to speak of the multiple risks to which the planters were exposed .^10^

These two languages barely meet, even though some readers might perhaps have wondered if the impassioned plea to understand the tyrannies suffered by the planters might not also sound curiously applicable to the situation of the population they held in thrall. This white discourse of freedom and slavery was not connected by Long to the slavery of Africans on the plantations.^11^ Indeed, for him, as for others, such a clear demarcation was critical to the maintenance of colonial power. A practice of *splitting*, holding contradictory thoughts separate, seemed essential. We emphasize the term ‘splitting’ since, later on, Freud and his followers would have much to say on its functions, most notably as a way of structuring experience, and providing a form of defence, in and beyond the earliest stages of psychic life.^12^

If we ask what is going on in Long’s text, or, to put it another way, if we wonder what might be the logic of Long’s argument, in its conscious and unconscious dimensions, various answers are possible. One interpretative strategy for the historian is to draw out the explicit or implicit functions of the polemic that Long is engaged in with regard to other people or institutional groupings around him; above all, in this case, his need to justify slavery in the face of metropolitan critiques. The historian’s task includes exploring the reasons for the timing of a text such as this, and teasing out its causes, purposes and effects, as a public intervention.

Long’s *History* was launched into a world of competing discourses about the nature and future of slave society. To understand, historically, the meaning, purpose, causes, and consequences of Long’s work entails considering, for instance, a variety of questions and assumptions about race, property, freedom, environment, nature, climate, gender, and human sensibility, peculiar to that era. One further step, we suggest, might be to bring psychoanalytic understandings into dialogue with more conventional forms of historical analysis. The historian might ask questions about what Long is engaged in here, by considering his writing both as his attempt to convince others, and as an effort to resolve his own doubts and defend himself against his own perceptions or knowledge. We might think about the writing as an exercise in the quelling of doubt, or in an unconscious ‘need’ for denial, not merely the conscious rehearsal and articulation of a given political position with which he was personally comfortable.

To pursue that latter line of interpretation immediately opens up questions, especially, if we want to envisage an unconscious process at work in an argument that he or any other long dead character may have had with themselves. Some historians in the past have ventured into bold speculation about the unconscious minds of the dead, treating historical actors as though patients on the couch. ‘Wild analysis’ is a temptation perhaps best avoided, as Freud himself showed, and also as he warned.^13^ On the other hand, even without claiming to ‘psychoanalyse’ the late Edward Long from afar (there is, after all, so very much of, and about, him we do not even begin to know), we may find value in adopting the vocabulary and the modes of thought that psychoanalysis has provided, for instance regarding projection, introjection, splitting, identification, and denial; that is to say, using these tools to grasp the curious features and tensions at work in the text itself, or in the forms of discourse and conversation that he was engaged in, and thereby to ask more searching questions about what Long may have been doing in writing his *History*.

The historian is, of course, interested in what is said and left unsaid, and attentive to contradictions. Here, in Long’s project, we need to pay particular attention to the nature of the relationship between his convictions, and the flickers of doubt that occur in their midst. To go further, one might reflect on the silences and absences that may, perhaps, mark disturbances for him, albeit alert to how we may misguidedly impose our own assumed meanings on his. We have choices at that point: how far to focus upon the nature of possible internal – intra-psychic – arguments at work, as opposed to inter-personal arguments, in relation to which, most obviously, this text itself was directed. To what degree do we prefer to consider Long as not only in battle with his opponents but also at odds with himself? In common parlance, we speak of these internal psychological conflicts, when we say, for instance, that a person is ‘wrestling with their conscience’.

How far might the argument mounted in Long’s *History* be there not only to make a case in the world, but also to banish doubts for the writer? The writing then might be regarded as a task of rebuttal of several different kinds at once. For it was surely not possible, in this case, entirely not to know the profound injustice at stake, nor that men and women who faced such punishments as he witnessed, described and sanctioned, could not do so without suffering tremendous pain and torment, whatever his claims about their natural differences. He knew they were human, like him, and suffered, even as he insisted – violently – upon the victims’ brute nature, and their imperviousness to feeling.

Of course there were accounts at the time that insisted on different racial thresholds of pain, or that drew upon ideas about the potential purifying or edifying effects of chastisement. Theories were advanced postulating profound differences between and hierarchies of ‘race’ (anatomical, moral, intellectual). Indeed, Long played no small part in fostering them. But all of these assumptions were contested in that period, which was perhaps why Long and others redoubled their efforts to deny them. Moments of doubt in Long’s *History* are fleeting and can easily be missed. What is contentious, however, in such a case is not the identification of these moments of doubt as such but the potential Freudian reading of this conflicted text, and behind that of the conflict it reveals in the author. Should the historian opt to make inferences about the mind of the writer, and the unconscious dimension of that mind?

Freud, we might speculate, in coming at Long’s account, would have sought to remark upon his repression of the counter-knowledge available to him, his capacity to repudiate, disavow or deny, and thereby render unconscious that knowledge to himself. When successfully achieved, this process, Freud suggests, may then leave the subject in question apparently conflict-free. Yet according to this account, symptoms may well emerge, evidence of something awry, signs of the return of the repressed. Long seemingly resolved an argument about the cruelty and barbarism that the planters were meting out upon the enslaved by his insistence – his passionate declamations – that there are incontrovertible, and essential, racial gulfs that render these others less pained than ‘us’. ‘Their’ suffering is not like ‘ours’, and is not to be regarded with the same empathy as ‘ours’. Through recourse to these ‘facts’, Long absolved himself, as the historian Elsa Goveia argues, from feelings of uncertainty.^14^ The strategy was to assert his omnipotence and omniscience, a lordly manner of assuring the reader – and himself – that the sufferer’s suffering did not really count; they did not feel it; it was not suffering as ‘we’ understand it.^15^

The historian now cannot know for sure what Long may actually have felt ‘in his heart’. What we have are his texts. And these, clearly, had multiple intended functions in debates of his time. We also have many other relevant historical documents, which situate his life and his thought, both in Jamaica and England. Texts, intended to be fictional or otherwise, are not necessarily, of course, indices to the moral feelings, or hidden beliefs of the author. Certainly, we cannot assume that Shakespeare’s beliefs are those of his character, Macbeth, or that Philip Roth’s sensibility is congruent with Portnoy’s, although, of course, much criticism, historically, has been built on the naïve premise that the writing is a window to the soul of its author. Even in political and historical writing, authors may choose, or at least adopt, different voices and registers, deploying words in response to given situations and rhetorical requirements, or the dictates of particular genres. It would be wrongheaded to assume a simple and necessary concordance between people or positions described in a text and inner beliefs. Nonetheless we can still wonder about the connections between a certain mind, or state of mind, and the writing, and note the splits, silences, hesitations, stresses and occlusions, as here, in Long’s *History*, which may be suggestive, amongst other things, of a psychic struggle occurring.

It is important to emphasize that this possible Freudian reading (a work of denial or disavowal taking place *through* the argument), need not depend upon assuming, anachronistically, knowledge, or a language of human feeling and empathy, unavailable in the time Long was writing. Those registers were apparent at the time. He attempted to counter them himself. ‘When the planters have complained of violations done to their liberty, he wrote, ‘the enemies of the West-India islands have often retorted upon them the impropriety of their clamouring with so much vehemence for what they deny to so many thousand Negroes, whom they hold in bondage.’ His answer was that ‘the higher estimation they put upon their own independence, the more indulgent masters were they to their slaves’.^16^ A psychoanalytic reading might focus upon the urgency and strain in this writing, as a mark, perhaps, of the author’s own motivated refusal to incorporate those various alternative forms of understanding, even though those forms were available then.

The insistence in the writing, that sense of redoubled effort when doubt creeps in, unease threatens, may have served to deal defensively with the counter-thesis of which Long was clearly aware: the all-too human connections between masters and slaves. Was there an unconscious identification by Long with the enslaved? Perhaps. He sometimes drew upon the language of slavery, as he suggested free-born Englishmen, such as himself, were also enchained. But that human connection itself between the planter class and the slaves who worked the plantations, seemed an affront to him; the text vehemently works to put the matter to rest. Any legal challenge to the planters’ assumed entitlement to possess ‘their’ slaves was, meanwhile, taken by Long as an outrageous abuse. In his polemic Lord Mansfield was accused of ‘the art of *washing the black-a-moor white*’.^17^ His *History* might be seen not only as making a case, but also providing an exercise in maintaining a kind of equilibrium, for writer and reader alike: it is about not allowing, not seeing, not hearing, as much as about encompassing, perceiving, knowing. Its function, perhaps, was to maintain a certain psychic economy, as well as a particular economic and social order, intact.

## CHOOSING OUR WORDS

Both Göring and Long, we might want to say, were in denial about the horrific nature of their actions. We discuss these two men as case studies, individuals who had powerful roles and identities, in respect of a wider group. They raise a specific question for us about the psychic mechanisms that enable individuals and groups to dehumanize others, and thus help to avoid the implications of their actions and to live with themselves. While the politics of remembering and forgetting have long been explicit concerns of historians, the particular emphases of these works have been varied. Research in recent decades, especially on nations and nationalism, has focused closely on what Pierre Nora called ‘les lieux de mémoire’ (sites of memory).^18^ Remembrance may consolidate an ‘imagined community’; so too, may occlusion and erasure – even major archives, of course, have sometimes been ‘misplaced’ or ‘lost’ in the service of national interests.^19^ Historians as well as therapists at times celebrate the achievement of recollection and commemoration – these processes may also be linked, at times, to forms of historical reparation – although we know too that remembrance does not only serve positive or emancipatory political ends. Memory, no less than forgetting, can be toxic, exploited in reactionary, defensive, grievance-ridden and nostalgic ways. Forgetting may be necessary, both individually and in groups, in order to function at all.^20^ Nations are required to forget in order to exist, the French philosopher Renan famously argued. Although to live only in the present may be a violent refusal of history, to live only in the past can be a horrifying attack on life, as Dickens memorably explored in *Great Expectations*.

In focusing on denial we aim to explore particular forms of remembering and forgetting, connecting and disconnecting, that can be made use of by individuals, institutions or indeed states.^21^ Denial, even in the specific sense of the quelling of an internal conflict, is not some exclusively modern concept, nor the preserve, alone, of Freud and his followers. Johnson’s *Dictionary* of 1755 glossed denial to mean negation, refusal, or even abjuration; the latter defined as the contrary of an acknowledgment of adherence. Johnson also included an entry for the term ‘denier’, meaning a contradictor, an opponent, one that holds to the negation of a proposition, but also potentially a ‘disowner’, ‘one that does not own’ or acknowledge, or even a ‘refuser’, ‘one that refuses’. As Johnson famously noted when reflecting on the demands of American colonists for independence from Britain: ‘How is it that we hear the loudest yelps for liberty among the drivers of negroes?’^22^ Statements of denial then, as now, can be taken many ways. The word itself may signify assertions that something is not happening, does not exist, is not true, or is not known about. It might indicate insistence that something that is said or believed is false. It may also mean disbelief in the existence or reality of a thing, incredulity about a natural, social or economic phenomenon (‘climate deniers’, of course, or, to cite another phrase that became a commonplace part of British political discourse in the 2010s, ‘deficit deniers’). In law it can refer to a refusal to acknowledge the validity of a suit.

In one sense the concept at stake in this essay is modern: ‘in denial’, according to the *Oxford English Dictionary* became a particular American buzz phrase and then passed into general usage later in the twentieth century. As it drily puts it: ‘ Now also in more general use, esp. in phr. *in denial* (orig. and chiefly U.S.)’. ‘In denial’ depends upon the psychoanalytic understanding but this is now assumed as a common-sense idea. We are talking, in that sense, of a repression, where knowledge of some kind is actively removed from the subject’s awareness, rendered unconscious, when new circumstances, say, the death of a loved one, particular passions, envy for instance, or drives, sexual in nature, for example, come into play, and cause the subject a psychical problem. A number of the examples amassed in the dictionary point to this particular psychoanalytic aspect of the term: so, for example, a reference to an explanation of ‘denial’, offered by Otto Rank, one of Freud’s followers, in a book, *Mental Hygiene* (1927) or, more recently, a reference to a work by the Kleinian analyst, Hanna Segal, in which she comments upon a particular case in 1979: ‘The denial of his mourning is also apparent in his running away.’

We can know something unconsciously, according to such accounts of denial, even as consciously we may operate sweetly innocent of the knowledge, just as we can walk a pavement oblivious of the ruins of dwelling places submerged beneath our feet. Freud believed, however, that this buried, or repressed, knowledge returned in various guises. A person might be adamant, for instance, not only that they behave faithfully to their spouse, but also that they *feel* wholeheartedly loyal and committed, even as that certainty may be belied by slips of the tongue and the pen, dreams, or everyday bungles. A patient may profess enthusiastic devotion to the project of their analysis, even as they arrive, repeatedly, very late, and forget to pay the bill. Freud, of course, dramatically and controversially ushered in a psychology based upon the idea that mind is always conflicted, and that we actively attempt to rid ourselves of certain mental contents, for example sadistic or masochistic propensities. The body too may speak another unconscious story: thus Freud described a hysterical patient who seemed to know nothing of sexual desire, yet whose hands conveyed a different drama: the one unbuttoning her clothes, the other doing them up.

Freud suggested how, from our infancies onwards, we are torn between ‘the pleasure principle’ and ‘the reality principle’. He built complex theories around both of these notions. In common parlance, too, this is recognized, when we speak of someone preferring to maintain a state of ‘blissful’ ignorance. Denial may signify the conscious knowing sacrifice of one’s own wants or desires (as when someone might say, ‘she decided to deny herself chocolate’); or – and here the *OED* definitions of the term bring us back to our particular story – in Freudian psychology, ‘an unconscious defence mechanism used to reduce anxiety by denying thoughts, feelings, or facts that are consciously intolerable’ (as, we are suggesting, with Edward Long).

There are a variety of other associated terms and ideas too which are deployed by historians with reference to how we may organize a field of vision or hearing, in order not to see or pick up the most disturbing element. Or, how we may banish an uncomfortable thought from our own conscious awareness. Some historians have made use, for instance, of *amnesia* – loss of memory, or *aphasia* – loss of the faculty of speech, to make a more complex point about willed unknowingness.^23^ Much was made too, in the 1960s and after, by psychologists and others, of the so-called ‘bystander effect’. The literature on that subject was fuelled in part by the astonishing case of Kitty Genovese, a woman murdered in 1964 in New York: a substantial number of bystanders who heard or saw ‘something’, chose not to intervene or report the event. Each, apparently, would assume inaction was appropriate, or surplus to requirements (a ‘diffusion of responsibility’). Psychoanalysts might have added to this the notion of ‘rationalization’. Faced by the murder occurring, either, it seems, people chose to believe that others would deal with the matter, or they concluded no action was needed, given that neighbours ‘reassuringly’ were not reacting either. Another useful concept, ‘psychic numbing’ (also developed in the 1960s) was Robert Jay Lifton’s means of describing, in the first instance, with regard to survivors of the Hiroshima atomic bombing, how traumatized people may manage to ‘turn off’ their emotions, cease to feel, undergo a ‘paralysis of mind’. Moreover Lifton observed how he himself, a researcher conducting fieldwork in Hiroshima and interviewing those directly affected, found himself pulled toward the same state of mind, a ‘selective professional numbing’; he went on to consider how other, more subtle forms of psychic numbing may operate, in less catastrophic environments too, as a means of screening ourselves from ‘the bombardment of stimuli,’ in everyday life.^24^

Disavowal is the refusal to avow, the disclaiming of responsibility or knowledge of something. We stress this term here since we argue it may often be more helpful, in historical analysis, to make use of this distinct concept, rather than just invoke the more ubiquitous ‘denial’. However, the fact that we are often struggling to find the most salient term may also say something about the ambiguity and slipperiness of the processes we are trying to capture and the uncertainty about whether the process is conscious or unconscious. Disavowal can be linked to the notion of a ‘blind eye’ or the rejection or rebuttal of something in plain sight, so carrying the implication of knowing and not knowing all at once. Freud had made use of the verb *verleugnen* to refer to the mental act of rejecting a perception as inconceivable, for instance in the *Introductory Lectures on Psychoanalysis*. James Strachey translated this as ‘to disavow’. In a later paper on *Fetishism,* Freud argued that disavowal did not erase the idea or perception in question, but rather the meaning, so that it could be understood as a suspension of the function of judgement. For him the term was associated with the disavowal of absence, whether of women’s lack of a penis or the death of the father. It has also been used, and widely so now, much more loosely to signify a refusal to think, a propensity to simply put aside and park what cannot be integrated, thus ignoring painful evidence.^25^

Freud’s ideas about conflict and repression were greatly elaborated by a second generation of followers, amongst them Melanie Klein, Wilfred Bion and Jacques Lacan, who, in different ways, explored further the way we may misrecognize ourselves, avoid pain, bury our guilt, and disclaim our desires. Lacan’s famous reading of a story by Edgar Allen Poe, ‘The Purloined Letter’, zeroed in upon an object, the epistle in question, hidden in plain view, on a mantelpiece where nobody (except the alert detective) could see it.^26^ Hence the casual leaving of a secret in an accessible location may turn out to be, by and large, a brilliant hiding place. As historians are well aware, archives may be technically open, but nobody bothers to look in them for reasons that might include, amongst others, an unacknowledged discomfort at the thought of what they contain. And as psychoanalysts and their patients often discover, something may in fact be in full view and yet not consciously seen at all. A patient may complain, for instance, that the analyst reveals nothing of herself, yet pass by for ages, failing to notice the analyst is wearing a wedding ring. ‘I didn’t know’, ‘I didn’t see’, can sometimes be so insistent that we wonder how was it possible, a kind of negative hallucination. The process may also be partial – a pushing aside of a thought to the margins, as it were, rather than into oblivion. Denials and evasions may be subtle or gross, and, at times, both parties to the analytic encounter may be drawn in, acting together in defiance of reality and actual perception. An analyst may only realize later, with the help, say, of a supervisor, some glaring failure on their own part to notice their own participation in an ‘enactment’. That term refers to the way the analyst may enter, unwittingly, a role in the patient’s own unconscious script, playing out a part: for instance, the patient’s requirement to be treated harshly and dismissively, or especially emolliently, and with ‘kid gloves’.

A follower of Freud’s who proved especially influential in looking at how groups can act in denial of reality was Bion. He was alert to how a group might silently and collectively ‘agree’ not to notice, as it were, some particular elephant in the room, or (to borrow an image from a famous tale) an emperor with no clothes. He came to differentiate what he called ‘work groups’, which are able to function more thoughtfully and creatively, from ‘basic assumption groups’ that are dominated by schizoid and paranoid mechanisms. Groups may operate to share in the radical distortion of reality, even its ‘scotomization’ (the creation of a total mental blind spot). A group, he postulated, may oscillate between such states, just as may an individual. His clinical descriptions provided rich examples of how splitting, projection and idealization (not least of himself as the putative leader of the assembled gathering, the one supposed to do the thinking for everyone) may abound in the life of groups, and, indeed, institutions.

Bion’s work may be of particular use in considering how institutions in the past can have been constructed and maintained in order to fail to see and to know.^27^ Attention to such considerations as corruption, public reputation, the exercise of power, or the pursuit of material interests is vital; but taken alone, these modes of explanation will often not suffice as we can see for example in the seemingly endless new revelations, over the last decade, of the sexual abuse of countless children by Catholic priests around the world. These individual exposés, it is now all too apparent, were always dwarfed by the deeper, scandalous story: the systematic hushing up of the evidence. The problem of abuse, it turns out, was rooted in the system, a state of active not knowing, repressing, even as the claim is always about ‘learning lessons’, getting rid of a few ‘rotten apples’. This litany of refusals, moving up the chain of command, is captured well in *Spotlight* (2015), a film about the endeavours of journalists at the *Boston Globe* some years earlier to follow the trail of abuses of children right up to the top.

Theodor Adorno merits particular attention here too in light of his attempts to think about the mass psychology of fascism and to explore the combined state of knowing and not knowing. As he surveyed the catastrophe of interwar German history, Adorno imagined political subjects who did not really believe what they claimed to believe. They had to perform allegiance to the idea that Jews were the devil incarnate and that ‘the Final Solution’ was necessary. Since they also knew this to be false, their performance was particularly frantic. ‘If they would stop to reason for a second’, Adorno wrote, ‘the whole performance would go to pieces, and they would be left to panic.’^28^ They were acting as though players in a drama, performing their enthusiasm, their identification with the cause, and not only for others, but also for themselves. They could not afford – psychologically – not to.

A different connection can be drawn here to Hannah Arendt. She was no disciple of Freud, yet we can make a link between Freud’s ‘denial’ and ‘disavowal’ and her attention to the concept of thoughtlessness, characterized in part by the absence of internal dialogue. This evacuation of feeling and disconnection of thought had a vital function in acquitting the subject (at least in their own eyes) from responsibility.^29^ She saw the repetition of empty and trivial truths by officialdom as a key characteristic of modern times. Her judgement of Eichmann, one of many aspects of her account that proved influential, and highly contentious, was that he was unable to think and to question the mass killing, accepting it all as a patriotic necessity, the banal executive of evil.^30^ Blind bureaucrats, thought-free, automaton-like apparatchiks, rather than individual sadistic monsters, represented, for her, the most terrifying agents of totalitarianism in the mid twentieth century.

That insistence in Arendt on states of mindlessness, and thoughtlessness, has powerful echoes in some of the more recent psychoanalytic work that is associated with denial and disavowal. Take here the useful vocabulary suggested by the psychoanalyst John Steiner, who explores the psychological dynamics of ‘turning a blind eye’. Steiner begins by reminding us about the many ways we may distort and misrepresent reality, and uses the story of Oedipus to examine a situation where there is access to reality but it is ignored for reasons that then require analysis. ‘I refer to this mechanism as turning a blind eye’, he writes, ‘because I think this conveys the right degree of ambiguity as to how conscious or unconscious the knowledge is.’ He is interested in the territory of disavowal, for example highlighting those ambiguous situations where we may have a vague awareness of choosing not to look at facts yet all the same proceed to evade that awareness. These evasions can lead to a variety of manoeuvres ‘which deny or conceal what has happened by creating a cover up’.

Freud had insisted that Oedipal impulses are part of everyone’s reality, however radically that knowledge is denied. In phantasy, he assumed, we have all sought to have total possession of our mothers, and to be rid of our fathers, and *vice versa*. Lacan suggested how ‘foreclosure’ of this fundamental and conflicted triangular situation produced a psychotic state of affairs. Klein, in a different register, offered acute reconstructions of infantile experiences, including wishes to devour and possess, the terror of being annihilated, the pain of loss and guilt, the possibility of mourning and reparation. Through the pain of recognition, of reparation, and of mourning, she suggested, when all goes well, growth occurs, and learning from experience may take place. If the Oedipal ‘crime’ is not even acknowledged but rather is covered up, as Steiner elaborates, evasion of reality will be damaging. He draws attention to the social and political implications of turning a blind eye and the dangers that result from this. As Steiner comments, ‘the fact that we do sometimes face the truth however imperfectly, is a considerable achievement’.^31^

Freud investigated how we make use of particular compromise ‘solutions’ such as fetishism or hysteria, to deal with thoughts at one remove. He was interested in the knowledge we do not allow ourselves to know, or can only know under certain conditions. Thus he mapped how we split, disavow, deny, or simply negate in order to maintain a certain psychic balance. In one example, Freud remarked that a person may heatedly and absolutely refuse a suggestion, even as, say, that person’s dream and association says ‘yes’. Taking the example of asking a patient who the person in their dream might be, he records the patient replying vehemently ‘It's *not* my mother’. When Freud then declares, ‘we emend this to: “So it *is* his mother”’, we might argue that he sounds too adamant, too quick to impose the contrary view, negating the negation all at once. But the crucial issue here (and of course he was not the first, Shakespeare, amongst others, having made the point clear enough before) is that at times we may ‘protest too much’ about our ignorance, or our innocence. ‘In our interpretation’, Freud remarks, ‘we take the liberty of disregarding the negation and of picking out the subject-matter alone of the association. It is as though the patient had said: “It's true that my mother came into my mind as I thought of this person, but I don't feel inclined to let the association count”.’ He further suggested that the content of a repressed image or idea could sometimes make its way into consciousness precisely on condition that it is negated. *‘*Negation’, he writes, ‘is a way of taking cognizance of what is repressed.’ So we have ways of half knowing, half allowing a thought in, on condition that it is simultaneously negated, or perhaps projected into something or someone else – given ‘house room’, so long as it does not implicate ‘me’.^32^

Our focus is on the unconscious processes associated with denial and disavowal. We are fully aware of the dangers of imposing a timeless human psychology. As historians, we see our task as reconstructing and exploring, not simply taking for granted, or imposing, our own systems of thought, social mores, rituals, or vocabularies of feeling, on other times and places. We require a history of the emotions, and to recognize that conceptions of what it is to be a person or a self, or indeed what it is to be human, have changed, often radically, over time. Yet for all that, we may still want to ask how much awareness may be shared, as to our basic ‘human’ propensities, in other historical conditions than our own. A great deal is shared, across modern times, to be sure. When we talk of the Nazis’ systematic endeavours to dehumanize Jews, or later, in many instances, to disavow knowledge of what took place – we assume an uneasy awareness on their part of the knowledge that the millions of people subjected to this dehumanizing, and then murderous treatment were in fact fully human, just like themselves. That knowledge was actively attacked, undone, dismantled, denied.^33^

Crucial for the historian is to see how a work of denial can operate institutionally as well as individually. For example, as we prepared this article early in 2017, the scandalous denial of the abuse of boys in the football world in Britain dominated the news. The contortions and evasions in subsequent official responses to such scandals soon became the central story. The explanations that we read here are revealing, and reminiscent at times of Freud’s famous account of a man’s evasion of his responsibility for a broken kettle. This marvellous observation is described by Freud during his exploration of a particularly troubling dream of his own – Irma’s Injection – in *The Interpretation of Dreams*:

The whole plea – for the dream was nothing else – reminded one vividly of the defence put forward by the man who was charged by one of his neighbours with having given him back a borrowed kettle in a damaged condition. The defendant asserted first, that he had given it back undamaged; secondly, that the kettle had a hole in it when he borrowed it; and thirdly, that he had never borrowed a kettle from his neighbour at all. So much the better: if only a single one of these three lines of defence were to be accepted as valid, the man would have to be acquitted.^34^

We may well all be prone to adopt broken-kettle logic at times. Admittedly, Freud’s kettle story, with its focus on avoiding small yet inconvenient truths in everyday life, may sit uneasily with the horrific nature and scale of the violence, killing and physical abuses to which we have referred in the pages above. But it points, once again, in a different vein, to the nature of psychological evasions and occlusions of reality, associated with denial.

Historians in the last few decades have charted the multiple orders of obfuscation and rationalization that have made it difficult, sometimes impossible, to study the data (located in secret or forgotten files) that would enable a due reckoning with the full scale of atrocities committed in the course of the European empires. Think of the work that has been done to explore the policies and practices that were used to suppress dissent and stifle insurgencies in imperial contexts (in Algeria, or Kenya, for example); to reckon with the work of the torturers; to face the reality of thousands of ‘disappearances’ (euphemism for murders), and arrangements for transitional justice in Latin America; to map the causes and consequences of genocide in Rwanda, and the attempts to wipe out that history; to confront the nature of ‘ethnic cleansing’ (another grotesque euphemism) in Bosnia. Work in each of these domains involves reconstruction of actual events, and the history of contemporaneous and subsequent cover-ups and denials. We have a rich literature too (history, fiction, memoir) on the creation, development and aftermath of apartheid and the politics of denial in South Africa; the processes of collaboration in occupied France, and its massaging away in many postwar accounts of that time. Or take the still opaque decision-making sequences that enabled Christian militia to slaughter many people in refugee camps in Lebanon in 1982, while the Israeli army remained unmoved on the sidelines. In the last of these instances, recall the difficulty of acknowledgement, both personally and collectively, of that history at all.^35^ How the work of memory comes up against the active work of forgetting is deftly conveyed in Ari Folman’s animated documentary, *Waltz with Bashir* (2008). Here is the story of Folman himself, seeking to overcome his amnesia, to recover his memories of what he witnessed as an Israeli soldier in the Lebanon War in 1982. The catalyst for his research is a dream concerning the night of the Sabra and Shatila massacres, a rich example of the ways in which the unconscious can point to a truth.

That denial and disavowal may beset organizations has been painfully apparent at points in the institutional history of psychoanalysis too.^36^ An organization may evolve ways of operating that spare its members the task of seeing and knowing, and above all, perhaps, of feeling, and then also acting appropriately in response. This was well shown by a pioneering group of psychoanalytic researchers in postwar Britain, who began to investigate how the culture of a particular organization could serve the psychic need of some or all of its members not to feel, say, overwhelmingly upset, guilty, or helpless. Doctors, for instance, might bypass the human dimension, and, when expected to bring bad news about incurable illnesses, or to deal with the painful upset of the patient, opt instead to leave such emotion-laden tasks largely or exclusively to the nurses.^37^

## A CASE IN HARINGEY, LONDON

In recent decades, a series of appalling cases about child and sexual abuse have highlighted the ways in which a range of professionals and institutions have failed to take care of vulnerable children and adolescents. Margaret Rustin’s reflection upon the Victoria Climbié Inquiry’s substantial report highlights the difficulties that social workers, health workers and the police experienced in dealing with perceptual evidence, their own mental pain in the face of it, and the ways in which they avoided it.^38^ Victoria, an eight-year-old who died in Haringey, in London, had been physically and mentally tortured at home over a long period. She had been seen by multiple agencies, none of which were able adequately to deal with what they saw, or to draw a firm enough line. Often it is only with hindsight that it seems possible for those involved to see where the lines were needed. Rustin draws on the notion of ‘turning a blind eye’.^39^ Although she analyses particular failings in this case, as did the official inquiry on which she bases her account, her deeper point is the ease with which any of us, in such circumstances, might be inclined to turn away, not through lack of feeling, but through the unbearable nature of the emotions stirred up. We all have defences against recognizing reality and this can involve severe distortions in the mind’s capacity to function.^40^

The Climbié Inquiry described organizations many of whose staff were under intense pressure. The people concerned, under-resourced internally and/or externally, struggled to cope and operated in such a way as to avoid the pain before them, creating protective structures. Individually and institutionally these can be understood as forms of ‘psychic retreat’. This term, helpfully proposed by John Steiner, describes a form of defensive organization (organization now applied to structures of mind itself) created by individuals who maintain a certain psychic equilibrium by severely titrating their access to reality.^41^ This may take neurotic or psychotic forms. Much of this builds on Freud’s own key insight that in psychosis the delusional system may be viewed not as the source of the madness, but as the patient’s attempt to create a ‘patch’ to deal with the sense of emptiness, devastation or fragmentation.

Workers in the services that were meant to be responsible for dealing with Climbié and her aunt seem to have been unable to notice what was going on, and therefore to think about it. Thinking required the prior act of holding in mind what is to be thought about. Social workers, for example, wanted to keep a distance from the intense horror of this mental and physical cruelty, and the madness. There was much comment in the report on the absence of written notes at a number of critical points – this act of *not* writing down was not only a function of the bureaucratic situation (harassed officials suffering time constraints, too much form-filling and so on), but also a more active mechanism to avoid the expression or even the knowledge of unbearable thought. Numerous examples of forgetting were subsequently documented, even the active destruction of evidence, all this pointing to the ways in which people were covering their tracks, for legal as well as for psychological reasons: unable to read and to tolerate the facts before their eyes. ‘The absence of thoughtfulness’, Rustin concludes, was evident both individually and at the level of systems. ^42^ It may be, then, that the failure to see what is in plain sight occurs because it would be too disturbing; we actively disconnect things, or attack the links between thoughts which logically belong together, because to connect them would be too agonizing. We may have varying internal resources to cope with our sight, or insight, but all of us, psychoanalysis would suggest, need support of some kind to bear confronting much reality at all. Here the current European refugee crisis comes powerfully to mind: bringing up the question not only of perpetrators and victims but of those whom Stan Cohen, in his insightful book, *States of Denial*, calls ‘bystanders’ (re-animating that 1960s literature, to which we referred earlier), or whom Michael Rothberg describes as ‘implicated subjects’.^43^

## CONCLUSION

Minds and institutions, in this schema, are seen as operating defences, more or less extreme, against unwelcome thoughts and feelings. We have suggested how an interpretation of Edward Long might require terms such as denial or disavowal. All white men, especially those ruling the plantations and presiding over a slave economy, lived with the intimate everyday knowledge of extreme violence. How did they ‘bridge’ between the notions of liberty and freedom that they cherished for themselves, and the awareness that ownership of the enslaved entailed horrific exploitation and cruelty? In Long’s case, we are arguing, even as that link was intimated, it was then actively – and violently – repudiated. Others such as Samuel Johnson could see the contradictions quite clearly and critiqued the hypocrisy. The development of antislavery sentiment in the 1770s made it increasingly difficult to ignore the view that colonial slavery was an iniquitous and cruel system. For planters, like Long, whatever their personal predilections, upbringing, or immediate milieu, it is clear that intellectual encounters with Enlightenment debates over the nature of man, raised questions, and invited a certain modicum of doubt about the basis of this system. Yet, at the same time, Long appears to speak as though entirely free of such doubt. To operate and endorse this system depended, for Long, upon refusal of full humanity to the slave, or even the consideration of that humanity, and a disregard for all of its inevitable detrimental consequences for bodily and mental health.

Long, we may surmise, managed to maintain an equilibrium through splitting: his life as a plantation owner, instigating as well as living close up to the horror, did not dismantle his defensive organization. He could not, or would not, see and connect. The need to disavow overrides everything. His conviction that Africans were essentially different was psychically and politically crucial to him, for reasons that included but were not restricted to material stakes (his vested economic interests). He managed to know and not know at the same time, in ways that enabled him, by and large, to secure his identity and place, providing a justification (financial, material, moral, ideological, emotional) for ‘business as usual’. In his account of the fauna and flora of Jamaica, for example, Long drew on the knowledge of Africans, yet he immediately undercut any recognition of his dependence on their expertise with an insistence that ‘brutes are botanists by instinct’. Negroes had a range of medicaments that worked, but they were incapable of theory. Their knowledge came from ‘the Creator …who has impartially provided all animals with means conducive to their preservation’. But this did not mean they were capable of rational thought.^44^

To consider how Long maintained such splitting leads us into psychological as well as socio-economic, cultural and political considerations, albeit in the knowledge that such personal investments, preferences and taboos are often obscure. That obscurity may arouse different reactions in the historian. Some, as we have noted, eschew all explicit exploration of the unconscious processes. Yet to assume that the external battle of ideas and obvious ‘vested interests’ in a given society is *all* that drives the person, is a presumption no less questionable than to assume that it is *only* affairs of the heart or forces in the unconscious that propel us to action. The question remains how to get access to the traces of that inner life, what to listen out for in the silences and the excesses, the contradictions and self-deceptions of speech, and with the aid of what kind of tools. The talking cure surely shows us not ‘what made everyone tick’ in the historical past, but how much we may blithely take for granted about motives and intentions, in writing histories of the dead.

To eschew explicit attention to the psychic life of the historical subject rarely, in fact, produces a psychology-free zone in history-writing: rather, as Peter Gay observed in his *Freud for Historians*, it may lead simply to implicit assumptions about the mind and behaviour, the resort to ‘common-sense’ notions about our driving human appetites, instincts and desires. Perhaps we might do well by recognizing, in light of Freud, the gaps themselves. And rather than claiming to grasp in full the psychological causes, we might be more modest: allowing ourselves some space to explore and to speculate, even as we insist upon the incompleteness of the picture, how much we simply do not know about motives. Thus hopefully we may aim at sounding less sure about fully accounting for people rather than more, with the benefit of that take on psychoanalysis. Yet, whatever one makes of psychic conflicts within individual people, denial and disavowal, we are suggesting, may operate equally powerfully in groups, networks, and institutions, providing opportunities for the historian to study these mechanisms.

The fact that it is modern to speak of people ‘in denial’, and owes a debt to Freud, does not mean it is inappropriate to apply it backwards, before the Freudian movement had emerged, and refashioned the concept, in order to capture moments where a subject, such as Long, is at odds with themselves, or where a group may ‘know’, and yet act apparently in ignorance of that knowledge, or in active defiance of it. It would be, as Quentin Skinner has observed, ‘an absurd self-denying ordinance’ to disallow concepts in historical interpretation that were not available to the actors themselves, where such concepts may throw a useful, additional light.^45^ That is a different issue, of course, from anachronistically assuming that very particular concepts, or, say, a specifically Freudian vision of the mind, were available to the historical actors themselves. On the other hand, psychoanalysts are also prone to forget, despite Freud’s own insistence that the poets had got there before him and that he was telling his readers what they already knew themselves, how much of the theory and folklore of the psychoanalytic movement existed in cognate forms long before.

* * *

Four years after publishing his *History,* Long wrote an extraordinary pamphlet: *English Humanity no paradox: or, an attempt to prove, that the English are not a nation of savages* (1778). In pondering the ubiquitous practice of flogging boys in schools he wrote, ‘The Rod is a dangerous weapon’ especially amongst those whose absolute authority can ‘cause violent bursts of fury … The hearts of some among them are steeled by habit against the compunctions of pity’. Some seemed to enjoy flogging too much and this had bad effects, driving them:

out of manly openness and sincerity, into all the mean subterfuges of low cunning, falsehood, treachery, and prevarication; sowing in tender minds the seeds of abject servility, cowardice, insolence, and every vile and despicable propensity. So that foreigners may well surmise, if any thing could subdue the generosity of our nature, it would be this unphilosophical, and slavish scouring for the posteriors for *seven or eight years* of our childhood.

He still dreamed, he continued, thirty years later, of being beaten. ‘May every Advocate for Tyranny be haunted *de die in diem* [daily], with these nocturnal visions, till he recants his error, and vows eternal enmity against all power unduly and rancorously exercised.’ ^46^

It seems that Long could not bear to know, what at another level he knew very well, the dominant place of the whip in his own psychic economy and on the plantations that he ruled, with all of their horror and barbarism.

## NOTES AND REFERENCES

### Footnotes

1 Leon Goldensohn, *The Nuremberg Interviews*, ed. R. Gellately, New York, 2004, p. 128.

2 The literature available on the psychological and economic reasons for climate-change denial is vast. Al Gore’s *An Inconvenient Truth: the Crisis of Global Warming*, New York 2004, and accompanying talks and films, proved especially influential. Numerous articles and popular books invoke this key word in titles such as *Climate Change Denial*, *Heads in the Sand*, *The Denial of Science*, or *Living in Denial.* For psychoanalytic reflections, see for example, *Engaging with Climate Change: Psychoanalytic and Interdi*s*ciplinary Perspectives*, ed. Sally Weintrobe, Hove, 2013; cf. Naomi Klein, *This Changes Everything: Capitalism vs. the Climate*, New York, 2014. At the same time, the psychological causes and consequences of Holocaust denial are also reinvestigated in recent books and films. *My Nazi Legacy* (2015), directed by David Evans, from a screenplay by Philippe Sands, and Sands’s book, *East West Street: on the Origins of ‘Genocide’ and ‘Crimes against Humanity*’ (London and New York, 2016), provide much food for thought regarding the politics and psychology of denial. Compare the remarkable story told in *The Flat*, a 2011 Israeli-German film co-production written and directed by Arnon Goldfinger. A feature film, *Denial* (2016), directed by Mick Jackson, based on a screenplay by David Hare, dramatized the case of David Irving, discredited historian of Nazi Germany, who in 1996 unsuccessfully sued Deborah Lipstadt and Penguin Books (the author and the publisher of *Denying the Holocaust*, 1993) for damaging his reputation by calling him a Holocaust denier. For the background, see Richard Evans, *Telling Lies about Hitler: the Holocaust, History and the David Irving Trial*, London, 2002; and D. D. Guttenplan, *The Holocaust on Trial: History, Justice and the David Irving Libel Case*, Cambridge, 2002. Among other salient films, consider Claude Lanzmann’s *Un Vivant qui passe* (1997) about Maurice Rossel, a Red Cross official who visited Theresienstadt during the Second World War and was, apparently, quite taken in by the model version of the camp he was permitted and wanted to see.

3 See Bruno Latour’s notable article, ‘Why has Critique Run out of Steam? From Matters of Fact to Matters of Concern’, *Critical Inquiry* 30, winter 2004, pp. 225–48.

4 Donald Trump’s counsellor Kellyanne Conway notoriously appealed to ‘alternative facts’ during a ‘Meet the Press’ interview on 22 Jan. 2017, when challenged about the President’s, and his press secretary, Sean Spicer’s, demonstrably false statements about the size of the crowds that had watched the inauguration. Evidence from photographs and urban transport statistics contradicted claims from the White House that attendance figures for Trump’s ceremony dwarfed the numbers on previous comparable occasions. The Orwellian aspect of these and other such pronouncements by the Trump team was much remarked and satirized at the time.

5 For a more extended discussion of Long, see Catherine Hall, ‘Whose Memories? Edward Long and the Work of Re-remembering’, in *Britain’s History and Memory of Transatlantic Slavery: Local Nuances of a ‘National Sin’*, ed. Katie Donington, Ryan Hanley and Jessica Moody, Liverpool, 2016, pp. 129–49.

6 Bryan Edwards, *The History, Civil and Commercial, of the British Colonies in the West Indies*, 3 vols, London, 1793, vol. 3, pp. 12–13.

7 Edward Long, *History of Jamaica*, 3 vols, London, 1774. In his discussion of the characteristics of ‘the Negro’ Long referenced Buffon, Linnaeus, Hume and Monboddo among others: vol. 2, pp. 351–83.

8 Long, *History*, vol. 2, pp. 446–7.

9 Long, *History*, vol. 2, fn. p. 458.

10 Long, *History*, vol. 1, pp. 4–5.

11 Edmund Morgan explored this paradox in *American Slavery, American Freedom: the Ordeal of Colonial Virginia*, New York, 1975.

12 Freud used the term ‘splitting’ to describe a phenomenon within the ego where two psychical attitudes co-exist without influencing each another, the one taking reality into consideration, the other disavowing it and replacing it by a product of desire (Jean Laplanche and Jean-Bertrand Pontalis, *The Language of Psychoanalysis*, London 1973, p. 427). Klein developed the term, exploring in detail the subject's capacity to split the object, and emphasizing how splitting serves from early on as a defence mechanism, in crucial ways, in all infantile development: ‘good’ and ‘bad’ aspects of an object, notably the breast, held completely apart, in phantasy. In ‘the depressive position’, Klein postulated that these different aspects of the object as well as of the ego, might be brought more closely together, with greater acceptance that ‘good’ and ‘bad’ elements are mixed (see *The New Dictionary of Kleinian Thought*, ed. Elizabeth Spillius, Hove, 2011, pp. 491–7).

13 Sigmund Freud, ‘“Wild” Psycho-Analysis’ (1910) and ‘Leonardo da Vinci’ (1910), *Standard Edition of the Complete Works of Freud* [SE] 24 vols, vol. 11. For subsequent debates about psychoanalysis and its cultural, political and historical applications, cf. David Stannard, *Shrinking History: On Freud and the Failure of Psychohistory*, Oxford, 1980; Peter Gay, *Freud for Historians*, New York, 1985; Daniel Pick, ‘Roma o Morte: Garibaldi, Nationalism, and the problem of psychobiography’, *HWJ* 57, spring 2004, pp. 1–33; Michael Roper, ‘Slipping out of View: Subjectivity and Emotion in Gender History’ *HWJ* 59, spring 2005, pp. 57–72; Stephen Frosh, *Psychoanalysis outside the Clinic: Interventions in Psychosocial Studies*, Basingstoke, 2010; *History and Psyche: Culture, Psychoanalysis and the Past*, ed. Sally Alexander and Barbara Taylor, Basingstoke, 2014.

14 Elsa V. Goveia, *A Study on the Historiography of the British West Indies to the End of the Nineteenth Century*, Mexico City, 1956, p. 60.

15 The downgrading and denial of suffering and violence are powerfully described in Susan Sontag, *Regarding the Pain of Others*, London, 2003; and Judith Butler, *Precarious Life: the Power of Mourning and Violence*, London, 2004.

16 Long, *History*, vol. 1, p. 5. For a contemporary critique of Long, see James Ramsay, *An Essay on the Treatment and Conversion of African Slaves in the British Sugar Colonies*, London, 1784, pp. 231–8.

17 Edward Long, *Candid Reflections upon the Judgement Lately Awarded by the Court of King’s Bench in Westminster Hall on What is Commonly called The Negroe Cause by a Planter*, London, 1772, Advertisement.

18
*Patriotism: the Making and Unmaking of English National Identity*, ed. Raphael Samuel, 3 vols, London, 1989; *Sites of Memory*, ed. Pierre Nora, 3 vols, New York*,* 1996–8; Emilio Gentile*, The Sacralization of Politics in Fascist Italy*, Cambridge MA, 1996.

19 David Anderson, ‘Guilty Secrets: Deceit, Denial and the Discovery of Kenya’s “Migrated Archive” ’, *History Workshop Journal* 80, 2015, pp. 142–60.

20 See, for example, David Rieff, *In Praise of Forgetting: Historical Memory and its Ironies*, New Haven, 2016. In the last year the campaign waged over Rhodes statues, first in Cape Town and then in Oxford and elsewhere, would be an obvious example of current debates which have provoked others to reflect on the necessity for forgetting. For an interesting early article see Eva Fairbanks, ‘How South Africa’s youth turned on their parents’ generation’, *Guardian*, 18 Nov. 2015.

21 See Stanley Cohen, *States of Denial*: *Knowing about Atrocities and Suffering*, Cambridge, 2001.

22 ‘Taxation no Tyranny. An answer to the resolutions and address of the American Congress’ (1775), *The Works of Samuel Johnson*, New York, 1913, 14 vols, vol. 14, p. 144.

23 See, for example, Ann Laura Stoler, ‘Colonial Aphasia: Race and Disabled Histories in France’, *Public Culture* 23: 1, 2010, pp. 121–56. Stoler’s essay, ‘Critical Incisions: on Concept Work and Colonial Incursions’, in *Duress: Imperial Durabilities in our Times,* Durham, N.C., 2017, has further relevant thoughts on these issues.

24 Robert Jay Lifton, *Witness to an Extreme Century*, New York, 2011, p. 128; cf. his *Death in Life: Survivors of Hiroshima*, New York, 1967, and *Hiroshima in America: Fifty Years of Denial*, New York, 1995.

25 Stuart Hall regards it as an essential concept for understanding the ways in which race has been ‘known and not known’ both in colonial Jamaica and in postwar Britain. See *Familiar Stranger: a Life between Two Islands*, London, 2017.

26 Jacques Lacan, *Écrits*, transl. Bruce Fink, London, 2006, chap. 2.

27 Wilfred Bion, *Experiences in Groups*, London, 1961.

28 Theodor Adorno, ‘Freudian Theory and the Pattern of Fascist Propaganda’, 1951, reprinted in *The Culture Industry: Selected Essays on Mass Culture*, ed. J. M. Bernstein, London, 1991, p. 137.

29 Hannah Arendt, *Eichmann in Jerusalem*, New York, 1963.

30 For the controversies, see *After Eichmann: Collective Memory and the Holocaust Since 1961*, ed. David Cesarani, London, 2013.

31 John Steiner, ‘Turning a Blind Eye: the Cover up for Oedipus’, *International Review of Psychoanalysis* 12, 1985, pp. 161–72. Reprinted in his *Psychic Retreats: Pathological Organisations in Psychotic, Neurotic and Borderline Patients*, London, 1993.

32 Freud, ‘Negation’ (1925), in SE*,* vol. 19, pp. 233–40, p. 235.

33 There is an extraordinary account of the process of dismantling and re-remembering in Gunter Grass, *Peeling the Onion*, London, 2007. On the struggle, during the 1940s, to establish ‘crimes against humanity’ and ‘genocide’ as shared, acceptable concepts in international law, see Sands, *East West Street*.

34 S. Freud, *The Interpretation of Dreams (*1900), *SE*, vol. 4, pp. 119–20. He later drew out the significance of the same ‘kettle’ story in his account of jokes. ‘This mutual cancelling-out by several thoughts, each of which is in itself valid, is precisely what does not occur in the unconscious. In dreams, in which the modes of thought of the unconscious are actually manifest, there is accordingly no such thing as an ‘either – or’, only a simultaneous juxtaposition’: *Jokes and their Relation to the Unconscious* (1905), SE, vol. 8, p. 205.

35 See, for instance, Avi Shlaim, *Israel and Palestine: Reappraisals, Revisions, Refutations*, London, 2009. (Questions of denial, remembering and forgetting run through these essays. Regarding Edward Said’s work, for instance, Shlaim suggests a common thread was to ‘make the case for a Palestinian presence in a world that tended to deny it’, p. 347.)

36 See, for example, Geoffrey Cocks, *Psychotherapy in the Third Reich: the Göring Institute*, New York, 1985; *Here Life Goes on in a Most Peculiar Way: Psychoanalysis Before and After 1933*, ed. Karen Brecht, Volker Friedrich, Ludger M. Hermanns, Isidor J. Kaminer, Dierk H. Juelich, Hamburg, 1995; Elisabeth Roudinesco, ‘Georges Mauco 1899–1988: un psychanalyste au service de Vichy. De l’antisémitisme à la psychopédagogie’, *L’Infini*, autumn 1995, pp. 73–84; Glen Gabbard, *Boundaries and Boundary Violations in Psychoanalysis*, New York, 1995; Aline Rubin, Belinda Mandelbaum and Stephen Frosh, ‘No Memory, No Desire: Psychoanalysis in Brazil during Repressive Times’, Psychoanalysis and History 18: 1, 2016, pp. 93–118; Dagmar Herzog, *Cold War Freud*, Cambridge, 2017, chap. 1.

37 A classic paper in this literature is Isabel Menzies-Lyth, ‘Social Systems as a Defence against Anxiety’ (1960), republished in her *Containing Anxiety in Institutions*, London, 1988, vol. 1. Cf. J. P. Martin with Debbie Evans, *Hospitals in Trouble*, Oxford, 1984; *Social Defences against Anxiety: Explorations in a Paradigm*, ed. David Armstrong and Michael Rustin, London, 2014.

38 Margaret Rustin, ‘Conceptual Analysis of Critical Moments in Victoria Climbié’s Life’, *Child and Family Social Work* 10, 2005, pp. 11–19.

39 Steiner, ‘Turning a Blind Eye’.

40 Bion, *Experiences in Groups*.

41 Steiner, *Psychic Retreats*.

42 Rustin, ‘Victoria Climbié’.

43 Cohen, States *of Denial*; Michael Rothberg is currently completing a book on ‘The Implicated Subject: Beyond Victims and Perpetrators’.

44 Long, *History*, vol. 2, p. 381.

45 ‘It would be a quixotic form of self-denying ordinance to insist that our language of explanation must at this juncture match whatever language the people in question applied or could have applied to themselves. … If we believe, for example, that Freud's concept of the unconscious represents one of the more important of these enrichments [in our stock of social explanations], we shall not only want to do our best to psychoanalyse the dead, but we shall find ourselves appraising and explaining their behaviour by means of concepts that they would have found, initially at least, completely incomprehensible’: Quentin Skinner, *Visions of Politics*, Cambridge, 2002, vol. 1, p. 50.

46 Long, *English Humanity no Paradox: or, an Attempt to Prove, that the English are not a Nation of Savages*, London, 1778, pp. 37–9.

